# Cardioprotective Effects of Coupled SH/NO System Modulators in a Rat Model of Doxorubicin-Induced Heart Failure

**DOI:** 10.3390/metabo16100715

**Published:** 2026-09-25

**Authors:** Victor Ryzhenko, Igor Belenichev, Olena Popazova, Nina Bukhtiyarova, Sergiy Oliynyk, Pavlo Petakh, Iryna Halabitska, Oleksandr Kamyshnyi

**Affiliations:** 1 Department of Medical and Pharmaceutical Informatics and Advanced Technologies, Zaporizhzhia State Medical and Pharmaceutical University, 69000 Zaporizhzhia, Ukraine; ryzhenko.victor@gmail.com; 2 Department of Pharmacology and Medical Formulation with Course of Normal Physiology, Zaporizhzhia State Medical and Pharmaceutical University, 69000 Zaporizhzhia, Ukraine; 3 Department of Histology, Cytology and Embryology, Zaporizhzhia State Medical and Pharmaceutical University, 69000 Zaporizhzhia, Ukraine; popazova.o.o@zsmu.edu.ua; 4 Department of Clinical Laboratory Diagnostics, Zaporizhzhia State Medical and Pharmaceutical University, 69000 Zaporizhzhia, Ukraine; buchtijarova.n.v@zsmu.edu.ua; 5 School of Medicine, Ewha Womans University, 52, Ewhayeodaegil, Seodaemun-gu, Seoul 03760, Republic of Korea; sergiyoliynyk622@gmail.com; 6 Department of Biochemistry and Pharmacology, Uzhhorod National University, 88000 Uzhhorod, Ukraine; pavlo.petakh@uzhnu.edu.ua; 7 Department of Therapy and Family Medicine, I. Horbachevsky Ternopil National Medical University, 46001 Ternopil, Ukraine; halabitska@tdmu.edu.ua; 8 Department of Microbiology, Virology and Immunology, I. Horbachevsky Ternopil State Medical University, 46001 Ternopil, Ukraine

**Keywords:** CHF, doxorubicin, metabolite cardioprotection, NO system, thiol-disulfide system, angiolin, thiotriazoline, heptral, N-acetylcysteine, mildronate

## Abstract

**Background:** The high prevalence of chronic heart failure (CHF) in industrialized countries, alongside its grave consequences—including increased mortality, disability, functional impairment, and reduced quality of life—underscores the urgent need to optimize core medical therapy. In this regard, metabolic cardioprotectants are of particular interest, as their action is aimed at interrupting or mitigating the cascade of adverse metabolic reactions and enhancing the compensatory and adaptive mechanisms of the heart. The aim of this study was to evaluate the effect of sulfur-containing pharmacological agents—namely, N-acetylcysteine (NAC), Heptral, Thiotriazoline, and Angiolin—on selected parameters of the NO and thiol-disulfide systems in the hearts of rats with experimental doxorubicin-induced CHF, followed by an assessment of their cardioprotective efficacy. **Methods:** CHF was modeled in 70 male Wistar rats weighing 180–190 g by intraperitoneal administration of doxorubicin at a cumulative dose of 15 mg/kg, divided into 6 injections over 14 days. The investigated drugs were administered intragastrically once daily as a suspension in 1% starch mucilage for 30 days following the 14-day doxorubicin protocol: Angiolin ([(S)-2,6-diaminohexanoic acid 3-methyl-1,2,4-triazolyl-5-thioacetate], 100 mg/kg), Thiotriazoline (morpholinium thiazotate, 100 mg/kg), NAC (100 mg/kg), Heptral (S-adenosylmethionine, 100 mg/kg), and Mildronate (250 mg/kg). In the rat myocardium, key components of the NO and thiol-disulfide systems—iNOS, eNOS, nitrotyrosine, stable NO metabolites, glutathione peroxidase-4 (GPX4), glutathione reductase (GR), reduced glutathione (GSH), and oxidized glutathione (GSSG)—were determined using enzyme-linked immunosorbent assay (ELISA), biochemical assays, and real-time PCR. In serum, cardio-specific biomarkers were measured, including the creatine kinase-MB isoenzyme (CK-MB), N-terminal pro-B-type natriuretic peptide (NT-proBNP), and soluble ST2 protein (sST2). **Results:** It was established that on day 45 following the 14-day doxorubicin administration, alongside a significant elevation in CK-MB, NT-proBNP, and sST2 levels (*p* < 0.05), there was a concurrent upregulation of iNOS mRNA expression, iNOS protein levels, and nitrotyrosine formation, accompanied by a downregulation of eNOS mRNA expression, eNOS protein levels, and stable NO metabolites (*p* < 0.05). Furthermore, heart tissue from CHF rats exhibited depletion of the glutathione pool within the thiol-disulfide system, characterized by decreased GPX4, GR, and GSH levels alongside increased GSSG concentrations (*p* < 0.05). Course administration of the test pharmacological agents to experimental animals post-CHF induction resulted in an improvement in the evaluated parameters of the coupled NO and thiol-disulfide systems, as well as overall cardioprotection. The study analysis demonstrated that Angiolin was statistically superior to Heptral and Mildronate, whereas Thiotriazoline and NAC outperformed Mildronate. **Conclusions:** These findings confirm the potential cardioprotection provided by modulators of the SH/NO system. Based on these results, further preclinical investigation of Angiolin as a promising cardioprotective agent for CHF is experimentally substantiated.

## 1. Introduction

Chronic heart failure (CHF) is the final stage of many cardiovascular diseases and a significant cause of disability and reduced life expectancy in developed countries. Over the past 20 years, the prevalence of CHF in developed countries has increased significantly, reaching epidemic levels in some. The high rate of early rehospitalization of patients with CHF is becoming not only a medical problem but also a societal one, as it entails significant economic costs for treatment. The importance of heart failure for the healthcare system will steadily increase [1,2,3]. Despite some successes, the problem of effective treatment of CHF remains one of the most complex and pressing issues in clinical medicine. Therefore, it is crucial to ensure that the results of clinical drug trials are as closely aligned with real-world practice as possible. Moreover, approaches to CHF treatment have changed significantly over the past 10 years. Adequate treatment of CHF at this stage requires the use of modern treatment regimens based on evidence-based medicine. The goal of drug therapy for CHF is to reduce mortality from this condition and improve quality of life [4]. Along with the basic therapy of CHF (beta-blockers, ACE inhibitors, diuretics, etc.), so-called metabolitotropic cardioprotectors play a very important role in the treatment of CHF [5,6,7]. Medications that can interrupt or reduce the cascade of adverse metabolic reactions caused by ischemia, collectively known as “metabolitotropic cardioprotectors,” exert a protective effect on the myocardium and hold clear promise in clinical practice. Currently, mildronate, trimetazidine, quercetin, thiotriazoline, and others have found clinical use as metabolitotropic cardioprotectors [8,9,10,11,12].

Currently, there is evidence of the involvement of NO in the multistage response of cardiomyocytes in CHF and the initiation of apoptosis, oxidative stress, and the progression of CHF. During ischemia, including myocardial ischemia, oxidative modification of low-molecular thiols is observed and, as a consequence, NO transport is impaired with the formation of its cytotoxic derivatives, which further enhance thiol oxidation. Currently, the concept of a coupled SH/NO system is known, a shift in which toward oxidized intermediates leads to nitrosative stress and the activation of signaling pathways of inflammation, apoptosis, and mitochondrial dysfunction [5,13,14,15,16]. Based on this, the role of thiol compounds and SH/NO system modulators as potential metabolite-active cardioprotectors becomes more apparent. For example, thiotriazoline is used in clinical cardiology and therapy for the treatment of angina [10]. The properties of the peroxynitrite and NO “trap” in the thiol antioxidant N-acetylcysteine are described [17]. Ademetionine (heptral), which is a precursor of cysteine, taurine, and glutathione and has NO scavenger properties, can be considered as a promising modulator of the SH/NO system [18]. Of interest as a modulator of the SH/NO system is the new molecule angiolin [19]. However, to date, the use of modulators of the SH/NO system, in particular acetylcysteine, heptral, angiolin, and thiotriazoline, in the treatment of CHF as metabolitotropic cardioprotective agents has not been justified.

**The aim of this study** was to investigate the effects of pharmacological agents containing an active sulfur atom in their structure—acetylcysteine, heptral, thiotriazoline, and angiolin—on certain parameters of the NO and thiol-disulfide systems in the hearts of rats with experimental doxorubicin-induced CHF, followed by an assessment of their cardioprotective effects.

## 2. Materials and Methods

### 2.1. Animals

Experiments were conducted on 70 male Wistar rats weighing 180–190 g, obtained from the vivarium of the Institute of Pharmacology and Toxicology of the Academy of Medical Sciences of Ukraine. The quarantine (acclimatization period) for all animals was 14 days. Upon receipt, each animal was examined by a qualified veterinarian to determine its health status. The animals were housed in polycarbonate cages measuring 550 × 320 × 180 mm with galvanized steel lids measuring 660 × 370 × 140 mm and glass drinking bowls. Each cage contained five rats. Each cage was labeled with the study number, species, sex, animal numbers, and dose. The cages were arranged on racks according to the dose levels and cage numbers indicated on the labels.

The following conditions were maintained in the animal housing room: temperature 20–24 °C, humidity 30–70%, lighting cycle 12 h light/12 h dark.

All rats were fed ad libitum a standard laboratory diet supplied by Phoenix, Ukraine. Municipal tap water, purified by reverse osmosis and UV sterilization, was provided ad libitum. Autoclaved alder (*Alnus glutinosa*) sawdust served as bedding. Animals that did not meet the criteria (sick, with a history of, or with concomitant illnesses such as depressive behavior, or those agitated under certain stressors) were excluded from the experiment for the duration of the quarantine period. Before the study, animals meeting the inclusion criteria were randomly divided into groups, numbered, and marked with a brilliant green solution. General animal appearance and clinical condition were monitored throughout the study.

#### Ethics Approval and Consent to Participate

All manipulations were carried out in accordance with the provisions on the use of animals for biomedical experiments (Strasbourg, 1986, as amended in 1998) and the “European applicable protection for vertebrate animals used for experimental and scientific purposes.” The experimental study protocols and their results were approved by the decision of the Bioethics Commission of ZSMPhU (Protocol No. 3 of 22 March 2025). All animal procedures followed the ARRIVE 2.0 guidelines (Supplementary Material).

### 2.2. Experimental Model

The doxorubicin model was used to reproduce chronic heart failure [20]. The doxorubicin pharmacological model of CHF can be considered the most effective, leading to the development of severe and progressive CHF in most animals. Administration of doxorubicin (intraperitoneally at a cumulative dose of 15 mg/kg, divided into 6 injections over 14 days) leads to a decrease in left myocardial contractility, eccentric remodeling, and the development of progressive CHF in rats [21].

### 2.3. Experimental Groups

Prior to the experiment, the rats were randomly divided into seven groups. The animals were randomized as follows: each of the 70 animals was numbered from 1 to 70 (marked on the back using a brilliant green solution). A randomized list of numbers from 1 to 70 was generated using computer software (Microsoft Excel for Microsoft 365, Microsoft Corporation, Redmond, WA, USA), and the resulting sequence was sequentially divided into blocks of 10 numbers. The following experimental groups were formed, with 10 animals in each group:

1. Intact group: healthy rats receiving 1% starch mucilage vehicle;

2. Control rats (animals with doxorubicin-induced CHF), which were injected with 1% starch mucilage;

3. Animals with CHF, which were injected with Angiolin, 100 mg/kg [19].

4. Animals with CHF that were administered Thiotriazoline, 100 mg/kg (Mukhina 2009).

5. Animals with CHF that were administered N-acetylcysteine (NAC), 100 mg/kg [22].

6. Animals with CHF that were administered S-adenosylmethionine (heptral), 100 mg/kg [23];

7. Animals with CHF that were administered the metabolic cardioprotector mildronate as a reference drug, 250 mg/kg [24].

An a priori power analysis was conducted based on the results of a pilot study. The significance level (α) was set at 0.05, and the type II error probability (β) was set at 0.20, corresponding to a statistical power (1 − β) of 0.80. The primary endpoint for sample size estimation was the iNOS level (ng/mL), with the minimal clinically meaningful effect (Δ) defined as its post-treatment change, accounting for inter-individual variability (σ).

The sample size was calculated using the following formula:

$$n\approx 2\frac{{\left(z_{1-\frac{\alpha }{2}}+z_{1-\beta }\right)}^{2}\sigma^{2}}{\Delta^{2}},$$

where $z_{1-\frac{\alpha }{2}}$ = 1.96- is the critical value for a significance level of 0.05,

$$z_{1-\beta }=0.84$$

is the critical value for a power of 1 − β = 0.80.

$$n\approx 2\frac{{\left(1.96+0.84\right)}^{2}0.29^{2}}{0.05^{2}}\approx 10$$

To evaluate the statistical power of the observed effect post hoc at a sample size of *n* = 10, Cohen’s d was calculated using the formula:

$$d=\frac{M_{1}-M_{2}}{S_{pooled}},$$

where $M_{1}-M_{2}$—difference in means, $S_{pooled}$ is the pooled standard deviation.

The obtained Cohen’s d value of 1.99 indicates a large effect size, thereby confirming the adequacy of the sample size.

All drugs were administered intragastrically once daily as a suspension in 1% starch mucilage using an atraumatic metal gavage tube for 30 days immediately following a 14-day course of intraperitoneal doxorubicin injections. The intragastric route provides ease of administration, high bioavailability, and corresponds to the anticipated oral administration used during treatment in clinical practice. Animals were sacrificed on day 45 after the first doxorubicin injection; under anesthesia, blood and heart tissues were collected for further analysis. A blinded method was applied at all stages: administration of test samples to the animals (test samples and starch mucilage were administered under codes from 1 to 7), as well as collection and analysis of biological material. Biological samples were numbered sequentially from 1 upwards. The research team conducting these procedures was blinded to the group assignments. Only the principal investigator had access to the decoding key. The final number of animals in the groups corresponded to the initially calculated sample size and was 10 animals per group. Precise weighing of the substances and chemical compounds was performed using a WT-500 torsion balance (Kyiv Medical Equipment Plant “Medapparatur”, Kyiv, Ukraine). Electronic micropipettes (Brand, Wertheim, Germany) and analytical glassware were used to prepare starch mucilage and solutions of drugs and pharmacological agents based on it. Animals were weighed using an AUX 220 electronic scale (Shimadzu Corporation, Kyoto, Japan) before each drug administration. Animals were administered intragastrically a volume of fluid calculated at 0.5 mL per 100 g of body weight.

### 2.4. Medications and Pharmacological Agents

The study included:

1. Doxorubicin “Ebewe” 50 mg/25 mL (EBEWE Pharma Ges.m.b.H. Nfg. KG, Unterach am Attersee, Austria);
2. S-adenosylmethionine (Heptral^®^), ((S)-5-(3-amino-3-carboxypropyl) methylsulfonio-5-deoxyadenosine) (Abbott S.r.l., Campoverde di Aprilia, Italy); N-acetylcysteine (NAC ^®^Hexal AG, Germany);
3. Thiotriazolin (morpholinium thiazate) PJSC “Kyivmedpreparat”, Kyiv, Ukraine;
4. Angiolin ([S]-2,6-diaminohexane acid 3-methyl-1,2,4-triazolyl-5-thioacecate) (synthesized at the Scientific and Technological Complex “Institute of Monocrystals” of the National Academy of Sciences of Ukraine (Series 2451117, Kharkiv, Ukraine));
5. Mildronate (2-(2-carboxyethyl)-1,1,1-trimethylhydrazinium) (Grindex (Latvia)).

### 2.5. Anesthesia and Euthanasia

At the conclusion of the experimental timeline, animals were humanely euthanized under general anesthesia, which was induced via intraperitoneal administration of sodium thiopental at a dose of 40 mg/kg. To prepare the anesthetic, lyophilized sodium thiopental (0.5 g vials; Kyivmedpreparat LLC, Kyiv, Ukraine; Code No. N01AF03) was dissolved in isotonic saline to obtain a 4% working solution. This solution was delivered using an insulin syringe at a volume of 0.1 mL per 100 g body weight. Following successful induction of deep anesthesia, spinal transection at the cervical level was executed to collect tissues for subsequent molecular and biochemical assays.

### 2.6. Collection and Processing of Biological Material

Whole blood was drawn from the abdominal aorta via syringe aspiration and transferred into specialized collection tubes with or without anticoagulants, depending on the required fraction. Serum and plasma separation was achieved by centrifugation at 1500 rpm (+4 °C) for 20 min using an Eppendorf 5804 R centrifuge (Eppendorf SE, Hamburg, Germany), after which the isolated fractions were stored at −80 °C.

The heart was immediately harvested and rinsed in a chilled 0.15 M KCl solution (+4 °C) at a 1:10 (*w*/*v*) ratio. Extraneous connective tissue, epicardial fat, main vessels, and intraluminal blood clots were excised before a second rinse in the same chilled KCl solution. The myocardial tissue was subsequently pulverized in liquid nitrogen to yield a uniform powder. Using a WT-500 torsion balance (Kyiv Medical Equipment Plant “Medapparatur”, Kyiv, Ukraine), 100 mg of the frozen tissue powder was weighed and thoroughly mixed with 10.0 mL of an ice-cold extraction medium (pH 7.4) comprising 250 mmol/L sucrose, 20 mmol/L Tris-HCl buffer, and 1 mmol/L EDTA.

To clear coarse cellular debris, the homogenate underwent primary centrifugation in a Sigma 3-30k refrigerated centrifuge (Sigma Laborzentrifugen GmbH, Osterode am Harz, Germany) at 1000× *g* (+4 °C) for 7 min. The harvested supernatant was transferred to fresh tubes and subjected to secondary centrifugation at 17,000× *g* (+4 °C) for 20 min using the same instrument. The resulting supernatant was collected and stored at −80 °C, while the dense mitochondrial pellet was resuspended for downstream assays.

For enzyme-linked immunosorbent assays (ELISA) and mitochondrial biochemical testing, the mitochondrial membranes were solubilized using Triton X-100. Specifically, 0.2 mL of a 0.1% Triton X-100 solution in 0.2 M Tris-HCl buffer (pH 7.4) was added to the pellet. The mixture was homogenized using a Potter–Elvehjem microhomogenizer (DWK Life Sciences GmbH, Wertheim, Germany) incubated on ice for 30 min, and then centrifuged at 24,000× *g* (+4 °C) for 20 min in the Sigma 3-30k centrifuge.

The apical myocardial region was fixed in Bouin’s solution for 24 h. Following standard dehydration through graded alcohols and clearing in chloroform, the tissue was embedded in Paraplast (McCormick Scientific, St. Louis, MO, USA). Serial 5 μm thick histological sections were cut using a Microm-325 rotary microtome (MICROM International GmbH, Walldorf, Germany), deparaffinized in ortho-xylene, rehydrated through ethanol series, and processed for real-time PCR applications.

### 2.7. Enzyme-Linked Immunosorbent Assay

Nitrotyrosine concentrations in both cytosolic and mitochondrial fractions of the heart homogenate were quantified via solid-phase sandwich ELISA using the Hycult Biotech, Uden, The Netherlands (Cat. No. HK 501-02) in accordance with the manufacturer’s protocol.

Endothelial nitric oxide synthase (eNOS) expression in the cytosol and mitochondria was measured using an enzyme immunoassay kit from Cloud-Clone Corp., Katy, TX, USA (Cat. No. PAA868Ra01). Similarly, inducible nitric oxide synthase (iNOS) expression was determined across both subcellular fractions using a MyBioSource kit (MyBioSource, Inc., San Diego, CA, USA; Cat. No. MBS023874). Glutathione peroxidase 4 (GPX-4) expression in cytosolic and mitochondrial samples was evaluated with the Rat GPX-4 ELISA Kit (MyBioSource, Inc., San Diego, CA, USA; Cat. No. MBS934198).

Systemic concentrations of myocardial injury markers were determined in serum samples: sST2 protein was measured using the Critical Diagnostics Presage^®^ sST2 Assay kit (Critical Diagnostics, San Diego, CA, USA; Cat. No REF# BC-1065), and N-terminal pro-B-type natriuretic peptide (NT-proBNP) levels were assessed using the Rat NT-proBNP ELISA Kit (Antibodies.com Europe AB, Stockholm, Sweden; Cat. No. A74911). All absorbance measurements were recorded on a SIRIO-S full-plate microplate analyzer (SEAC S.r.l., Calenzano, Italy).

### 2.8. Biochemical Methods [25]

Nitric oxide (NO) metabolite levels (NOx) in the cytosolic and mitochondrial fractions were quantified using the Griess diazotization method. Briefly, 1.0 mL of the harvested supernatant was deproteinized by adding 100 µL of 0.092 M zinc sulfate and 100 µL of 1 M sodium hydroxide. The reaction mixture was thoroughly vortexed and incubated for 30–40 min at room temperature. Following incubation, samples were centrifuged at 4000× *g* (+5 °C) for 10 min using an Eppendorf™ 5430 G centrifuge (Eppendorf SE, Hamburg, Germany).

A 100 µL aliquot of the resulting clear supernatant was transferred into a microplate well and mixed with 0.5 mM vanadium (III) chloride to convert nitrate to nitrite. Subsequently, 50 µL of sulfanilamide and 0.2 µL of N-(1-naphthyl) ethylenediamine dihydrochloride were introduced to yield a total reaction volume of 300 µL. After incubating the samples for 30 min at 37 °C, optical density was recorded at 540 nm. NOx concentrations were interpolated from a linear standard curve generated with sodium nitrate (0–50 µmol/L) and expressed as µmol/L of tissue fraction.

Glutathione reductase (GR) activity in both cytosolic and mitochondrial fractions was measured spectrophotometrically by monitoring NADPH oxidation rate at 340 nm. Assays were conducted in 10 mm quartz cuvettes containing 2.7 mL of 50 mM potassium phosphate buffer (pH 7.0) supplemented with 1 mM EDTA, 0.1 mL of 0.1 mM NADPH solution, and 0.1 mL of 0.5 mM oxidized glutathione (GSSG) solution.

The enzymatic reaction was initiated by adding 0.2 mL of the biological supernatant into the cuvette and mixing immediately. Absorbance changes were monitored over 5 min against a blank containing all components except GSSG. Specific activity was calculated and expressed as µmol/mg protein/min. Total protein content was quantified using the Quick Start™ Bradford Protein Assay kit (Bio-Rad Laboratories, Inc., Hercules, CA, USA). All optical measurements were performed on an Eppendorf BioSpectrometer (Eppendorf SE, Hamburg, Germany).

Reduced (GSH) and oxidized (GSSG) glutathione levels were measured fluorometrically in both cellular fractions based on the reaction of o-phthalaldehyde (OPA) with GSH to yield a fluorescent adduct (Ex/Em = 340/420 nm).

For GSH quantification, 0.1 mL of the supernatant and 0.5 mL of 1% o-phthalaldehyde were added to 2.0 mL of 0.5 M phosphate buffer (pH 8.0). The mixture was vortexed, incubated for 5 min, and fluorescence intensity was recorded at Ex/Em = 340/420 nm.

To determine GSSG content, 0.1 mL of supernatant was added to 2.0 mL of 0.5 M phosphate buffer (pH 12.0). To prevent interferences, reduced glutathione was masked by adding 0.04 mL of 0.5 mM 1-methyl-4-vinylpyridinium, followed by a 60 min incubation at room temperature. Subsequently, GSSG was converted back to GSH by adding 0.1 mL of an enzymatic reducing solution (38 units glutathione reductase and 7 mg NADPH dissolved in 20 mL of 0.5 M phosphate buffer, pH 7.4) and incubating at 37 °C for 2 min. Finally, 0.5 mL of o-phthalaldehyde was added, and fluorescence was measured at Ex/Em = 340/420 nm. Glutathione concentrations were derived from standard calibration curves. All fluorometric analyses were executed on a Cary Eclipse Fluorescence Spectrophotometer (Agilent Technologies, Inc., Santa Clara, CA, USA).

Creatine kinase-MB (CK-MB) isoenzyme activity in serum samples was evaluated using commercial diagnostic assay kits from High Technology Inc. (North Attleboro, MA, USA) according to the manufacturer’s protocol [25].

### 2.9. Polymerase Chain Reaction in Real Time

Transcriptional levels of inducible nitric oxide synthase (Nos2/iNOS) and endothelial nitric oxide synthase (Nos3/eNOS) were quantified by RT-qPCR using a CFX96 Real-Time PCR Detection System (Bio-Rad Laboratories, Hercules, CA, USA; Cat. No. 185-5096). Total cellular RNA was extracted from apex myocardial tissue using TRIzol™ Reagent (Cat. No. 15596026) in accordance with the manufacturer’s protocol. RNA concentration and purity were assessed spectrophotometrically, and complementary DNA (cDNA) was synthesized using the RevertAid™ First Strand cDNA Synthesis Kit (Cat. No. K1622). Specific primer sets for target and reference genes were designed and validated via NCBI Primer-BLAST (www.ncbi.nlm.nih.gov/tools/primer-blast, accessed on 1 June 2023) and synthesized by Thermo Scientific (Waltham, MA, USA): β-actin (Actb, 5′–3′) forward ACAACCTTCTTGCAGCTCCTC, reverse TCGTCATCCATGGCGAACTGG; iNOS (Nos2, 5′–3′) forward GTTCCTCAGGCTTGGGTCTT, reverse CCGTGGGGCTTGTAGTTGAC; and eNOS (Nos3, 5′–3′) forward CCCAGGAGAGATCCACCTCA, reverse CAGCACATCCTGGGTTCTGT. The amplification reaction was performed in a final volume of 20 µL containing 10 µL of 2× Maxima SYBR Green/ROX qPCR Master Mix, 0.5 µL of each gene-specific primer, 2 µL of cDNA template, and nuclease-free water. The thermal cycling conditions consisted of an initial denaturation step at 95 °C for 10 min, followed by 45 cycles of denaturation at 95 °C for 15 s, primer annealing at 60 °C for 40 s, and elongation at 72 °C for 40 s. SYBR Green fluorescence was measured automatically at the end of each elongation step. The β-actin (Actb) gene served as the internal reference for normalising target gene expression. Relative target gene expression was calculated using the comparative 2^−ΔΔCt^ method, with individual threshold values converted via 2^−ΔCt^ (where ΔCt = Ct target gene − Ct housekeeping gene) and subsequently log-transformed as Log2(relative expression). All reactions were run in triplicate.

### 2.10. Statistical Analysis

Statistical analyses were performed using STATISTICA 6.0 and the R statistical computing environment (version 4.6.1). Continuous variables are presented as mean ± standard deviation (SD). Normality of data distribution was assessed using the Shapiro–Wilk test. Differences between multiple experimental groups were analyzed using one-way analysis of variance (ANOVA). Pairwise comparisons were conducted using the two-sample independent Student’s *t*-test based on summary statistics with Bonferroni correction. Differences were considered statistically significant at *p* < 0.05.

## 3. Results

The conducted studies revealed that 30 days after the 14-day administration of doxorubicin, significant increases in molecular and biochemical markers of CHF—CK-MB fraction by 2.25 times (*p* ≤ 0.05), NT-pro BNP by 3.5 times (*p* ≤ 0.05), and sST2 by 5.25 times (*p* ≤ 0.05)—were detected in the blood of control rats compared to similar indicators in the intact group (Table 1). This indicates damage to cardiac myocytes, fibrosis, and deterioration and restructuring of the heart characteristic of CHF. Modeling CHF resulted in suppression of NO synthesis, which plays an endothelial- and cardioprotective role in myocardial pathology. Thus, in the cytosolic and mitochondrial fractions of the myocardium, a decrease in the concentration of stable nitric oxide metabolites by 40% (*p* ≤ 0.05) was observed, against the background of a decrease in the expression of endothelial nitric oxide synthase eNOS by 63.2% (*p* ≤ 0.05) and 42.1% (*p* ≤ 0.05), respectively, and an increase in the expression of its inducible form, iNOS, by 4 and 2.83 times, respectively (*p* ≤ 0.05), compared with similar indicators of the intact group (Table 2 and Table 3). In parallel, in sections of the apex of the heart of this group of rats, a decrease in the expression of eNOS mRNA by 89% (*p* ≤ 0.05) and an increase in the expression of iNOS mRNA by 7.8 times (*p* ≤ 0.05) were observed compared with the values of intact animals (Table 4).

In parallel, disturbances in the myocardial thiol-disulfide system were also recorded, the intermediates of which play an important role in the transport and bioavailability of nitric oxide. Thus, in the cytosolic and mitochondrial fractions of the myocardium of the control group of rats, suppression of the glutathione branch of the thiol-disulfide system was observed—a decrease in the activity of glutathione reductase (GR) by 67.8% (*p* ≤ 0.05) and 66.0% (*p* ≤ 0.05), GPX4 expression by 54.0% (*p* ≤ 0.05) and 67.5% (*p* ≤ 0.05), a decrease in the concentration of reduced glutathione by 73.8% (*p* ≤ 0.05) and 77.9% (*p* ≤ 0.05) against the background of an increase in the level of its oxidized form by 2.9 times (*p* ≤ 0.05) and 2.57 times (*p* ≤ 0.05) compared to the values of the intact group. These observations indicate that modeling CHF by administering doxorubicin suppresses the expression of eNOS, which is involved in endogenous mechanisms of cardioprotection, enhances the expression of iNOS, and also leads to depletion of the glutathione branch of the thiol-disulfide system, which together plays a major role in the implementation of the nitrosative stress program, as evidenced by an increase in cytosolic nitrotyrosine by 6.98 times (*p* ≤ 0.05) and mitochondrial nitrotyrosine by 4.37 times (*p* ≤ 0.05) in myocardial fractions compared to the intact group (Table 2 and Table 3).

A course of administration of the studied pharmacological agents to experimental animals after modeling CHF resulted in an improvement in the studied parameters of the coupled NO and thiol-disulfide systems, as well as in markers reflecting overall biochemical cardioprotection. Thus, administration of heptral to animals with CHF resulted in a decrease in the cytosolic expression of iNOS, an increase in the expression of eNOS, an increase in the concentration of NO Metabolites against the background of an increase in GR activity, GPX4 protein expression, and GSH concentration, along with a decrease in nitrotyrosine levels compared to the group of control animals (*p* ≤ 0.05). Heptral also increased the expression of eNOS mRNA and decreased the increased expression of iNOS mRNA in the apex of the heart of rats with CHF compared to the control group (*p* ≤ 0.05) (Table 5). Heptral did not have a reliable effect on the parameters of the nitric oxide system and the glutathione pathway of the thiol-disulfide system of cardiac mitochondria (Table 4 and Table 6). A course of heptral administration to rats with experimental CHF resulted in a reduction in cardiac damage markers—CK-MB by 21.3%, NT-proBNP by 27.2%, and sST2 by 26.3%—compared to animals in the control group (*p* ≤ 0.05), indicating the manifestation of metabolic cardioprotection by this pharmacological agent (Table 1).

A course of NAC administration to animals with CHF had a more pronounced effect on NO system parameters and the glutathione branch of the thiol-disulfide system, especially in cardiac mitochondria, than heptral. After a course of administration, NAC resulted in an increase in GR activity by 76.3 and 66.3% and GPX4 expression by 89.2 and 34.0%, an increase in GSH by 75.0 and 61.8% and a decrease in GSSG by 25 and 31.2%, respectively, in the mitochondria and cytosol of the myocardium of rats with CHF compared with similar parameters in the control group (*p* ≤ 0.05). Also, in the cytosol and mitochondria of animals with CHF receiving NAC, the expression of iNOS decreased by 38 and 46%, and the concentration of nitrotyrosine decreased by 52.5 and 54.3%, respectively, compared with the control (*p* ≤ 0.05) (Table 2 and Table 3). NAC also reduced elevated iNOS mRNA expression in the cardiac apex of experimental animals by 50.7% (*p* ≤ 0.05). NAC also increased NO metabolite concentrations in the cytosolic and mitochondrial myocardial fractions of rats with CHF by 35.3% and 38.8%, apparently due to an increase in eNOS expression by 76.8% and 41.3%, respectively, compared to control values (*p* ≤ 0.05), as well as a 5.25-fold increase in eNOS mRNA expression. Administration of NAC to rats with CHF resulted in a decrease in circulating biochemical and molecular markers of cardiac damage—CK-MB by 30.7%, sST2 by 50.8%, and NT-proBNP by 42.2% compared to the control group, which may indirectly indicate a biochemical cardioprotective effect of this pharmacological agent.

We were also interested in the drug thiotriazoline, which exerted virtually equivalent positive effects on the NO and glutathione systems.

Thus, administration of thiotriazoline to rats with CHF led to an increase in the concentration of NO metabolites in the myocardial cytosol and mitochondria by 41.2% and 51.4%, respectively, along with an increase in eNOS expression by 94.3% and 44.1%, respectively, compared to control values (*p* ≤ 0.05), along with a 10.5-fold (*p* ≤ 0.05) increase in eNOS mRNA expression in the cardiac apex of experimental animals (Table 2, Table 3 and Table 4). Thiotriazoline exhibited a more significant antioxidant effect than heptral and NAC, reducing the level of nitrotyrosine in the cytosol and mitochondrial fractions of the myocardium of rats with CHF by 65.6 and 63.5%, respectively, compared to the control (*p* ≤ 0.05). A similar effect of thiotriazoline on nitrosative stress is realized by a decrease in iNOS expression in the cytosol and mitochondria of the myocardium by 51.6 and 51.7%, and a decrease in iNOS mRNA expression by 67.6% in the apex of the heart of rats with CHF (*p* ≤ 0.05) (Table 2, Table 3 and Table 4) against the background of a positive effect on the indices of the glutathione branch of the thiol-disulfide system (Table 5 and Table 6). In the cytosol and mitochondria of the myocardium of rats with CHF that received thiotriazoline, an increase in GPX4 expression by 60.4 and 103.5%, increased GR activity by 70 and 97%, and a concentration of reduced glutathione by 77.3 and 107.4% were observed against the background of a decrease in the oxidized form of glutathione by 41.5 and 32.0%, respectively, compared with the control group (*p* ≤ 0.05). Thiotriazoline exhibited a more pronounced positive effect on the NO and thiol-disulfide systems and also decreased circulating biochemical and molecular cardio-specific markers in animals with CHF—CK-MB by 37.1%, NT-proBNP by 54.5%, and sST2 by 61.7% (*p* ≤ 0.05), which can be regarded as a manifestation of biochemical cardioprotection.

The novel molecule Angiolin appears to be the most compelling candidate in terms of metabolic cardioprotection (Table 1, Table 2, Table 3, Table 4, Table 5 and Table 6). Thus, in the blood of CHF rats treated with Angiolin, a decrease in biochemical and molecular markers of cardiac destruction—CK-MB by 47.1%, NT-proBNP by 60%, and sST2 by 73% (*p* ≤ 0.05)—was recorded compared to the control group. Administration of Angiolin to rats with CHF was found to significantly activate the glutathione branch of the thiol-disulfide system in the myocardium. Thus, in the mitochondrial and cytosolic fractions of the myocardium, under the influence of Angiolin, there was an increase in GPX4 expression by 152.6 and 90.2%, GR activity by 2.2 times and by 108.9%, an increase in GSH concentration by 2.7 times and by 188.6%, against the background of a decrease in the concentration of its oxidized form (GSSG) by 47.2% and 46.8% compared to the control values (*p* ≤ 0.05). Angiolin inhibited the activity of nitrosative stress in the myocardium of rats with CHF, reducing nitrotyrosine in the mitochondria by 72.7% (*p* ≤ 0.05) and cytosol by 70.8% (*p* ≤ 0.05) compared to the control values. This effect of Angiolin is apparently associated with both the activation of the glutathione branch of the myocardial thiol-disulfide system and the normalization of the nitrooxidative system in CHF. Thus, in the group of animals with CHF that received a course of Angiolin, a decrease in iNOS expression in the cytosol by 56.7% (*p* ≤ 0.05) and in mitochondria by 59.1% (*p* ≤ 0.05) was observed, as well as a decrease in iNOS mRNA by 76.1% (*p* ≤ 0.05) in the apex of the heart. Angiolin increases NO production (increase in stable NO metabolites by 58.8% and 57.8% (*p* ≤ 0.05) in the cytosol and mitochondria, respectively) due to an increase in eNOS expression by 171.2% and 75.0% (*p* ≤ 0.05) in the cytosol and mitochondria and an increase in eNOS mRNA expression by 19.5 times (*p* ≤ 0.05) in the apex of the heart compared to the group of rats with CHF without experimental therapy (control). Administration of the metabolic cardioprotector mildronate to rats with CHF did not significantly affect the parameters of the thiol-disulfide system and the NO system, with the exception of a significant effect on the nitrotyrosine indicator in the myocardial cytosol and the iNOS mRNA indicator in the apex of the heart of rats with experimental CHF (Table 2, Table 3, Table 4, Table 5 and Table 6). Nevertheless, a course of Mildronate administration led to a reduction in circulating biochemical and molecular markers of cardiac damage in animals with CHF—CK-MB by 21%, NT-proBNP by 32.1%, and sST2 by 25.4% compared to the control (*p* ≤ 0.05). Mildronate apparently exerts its cardioprotective effects through different mechanisms.

To determine the comparative efficacy of the pharmacological agents, pairwise comparisons (Student’s *t*-test) with Bonferroni and Holm adjustments for multiple testing were performed using the R software package (version 4.5.3; R Foundation for Statistical Computing, Vienna, Austria (Supplementary Material)). Statistical analysis revealed that Angiolin was superior to Heptral and Mildronate, and that Thiotriazoline and NAC were superior to Mildronate, without demonstrating a statistically significant difference across all other treatment pairs.

## 4. Discussion

Our current study on modeling the doxorubicin model of CHF is in line with our previous work and the studies of other authors [26,27,28]. A feature of the formation of the doxorubicin model of CHF, in contrast to other cardiomyopathies, is toxic damage to myocardial mitochondria, which makes the mitochondria a source of reactive oxygen and nitrogen species, proapoptotic proteins, which, against the background of severe energy deficiency, leads to the activation of oxidative and nitrosative stress and apoptosis of cardiomyocytes [5]. These molecular and biochemical mechanisms lead to cardiac remodeling and the development of heart failure. Oxidative and nitrosative stress, mitochondrial dysfunction, and apoptosis result in disruption of the contractile structure of cardiomyocytes, functional asymmetry, altered intercellular interactions, interstitial fibrosis, loss of myocardial fiber spiral organization, and changes in the shape of cardiac chambers [29,30]. In our case, when modeling CHF with doxorubicin, confirmation of these structural and functional disturbances in the heart (impairments in cardiac autonomic regulation, dysregulation of ventricular electrical activity, slowing of atrioventricular conduction, persistent diastolic dysfunction, as well as disturbances in cardiomyocyte morphofunctional parameters, including decreased cell density, reduced nuclear RNA concentration, and increased density of apoptotic cells) [15,31,32] was an increase in the concentration of sST2 protein in the blood of experimental rats compared to the intact group. sST2 (Suppression of tumorogenicity 2, Growth Stimulation expressed gene 2, stimulating growth factor expressed by gene 2, also known as IL1RL1) is a member of the IL-1 receptor superfamily. sST2 is a receptor for IL-33. sST2 is a marker of fibrosis and remodeling of cardiac tissue, released by cardiomyocytes and fibroblasts [33]. An increase in sST2 concentration indicates cardiac remodeling and the development of heart failure after 45 days in animals that received doxorubicin for 14 days [34]. We also recorded an increase in NT-proBNP, indicating myocardial damage characteristic of heart failure. NT-proBNP is elevated in left ventricular dysfunction and heart failure [35]. It is also known that NT-proBNP, sST2 and CK-MB are significantly elevated in the blood of patients treated with doxorubicin for cancer and who were subsequently diagnosed with heart failure [36]. In the doxorubicin model of CHF, there is a disruption of the electrical function of the heart (according to ECG data—a disruption of automatism, conductivity and contractility) and neurogenic mechanisms of its regulation (decentralization of neurogenic regulatory circuits, a decrease in the spectrum power and an increase in the stress index) can in this case be considered as a manifestation of diastolic dysfunction in experimental animals [32]. Our findings are consistent with current concepts of the mechanisms of molecular-biochemical disturbances, which are closely linked to changes in the nitroxidergic and thiol-disulfide systems of the myocardium. There is evidence that CHF is associated with decreased expression of eNOS, which is involved in endogenous cardioprotective mechanisms, and increased expression of iNOS, which plays a key role in the nitrosative stress program. Excessive NO production, coupled with increased iNOS expression and a deficiency of thiol antioxidants, leads to damage to the mitochondrial electron transport chain and may also be associated with the development of mitochondrial dysfunction in CHF. Mitochondria damaged in CHF subsequently become an important source of ROS and cytotoxic forms of NO. Mitochondrial NOS, like iNOS, is significantly activated in response to ischemia, mitochondrial calcium uptake, increased superoxide radical levels, lactic acidosis, inflammation, and increased IL-1β and TNF-α concentrations. However, NO molecules produced by iNOS are converted to peroxynitrite under the influence of ROS [5,30,37]. What was most interesting about our study was that, in parallel with the disruption of the NO system, a disruption was also recorded in the myocardial thiol-disulfide system, the intermediates of which play an important role in the transport and bioavailability of NO. It is well known that NO is an unstable, short-lived radical, and its stabilization and subsequent transport are facilitated by mechanisms such as the formation of stable S-nitrosole complexes with thiol-containing low-molecular compounds (glutathione, cysteine, methionine) [38,39]. Under conditions of deficiency of thiol compounds (oxidative stress, ischemia, intoxication, hypertension, etc.), NO transport is disrupted, since nitrogen monoxide is attacked by ROS such as superoxide radical and hydroxyl radical, converting into a cytotoxic product—peroxynitrite [16,40]. A deficiency of low-molecular-weight thiols leads to increased nitrosylation reactions of the Cys sites of the mitochondrial inner membrane antiporter protein, leading to increased polarity and permeability to ions and proteins, which leads to the initiation of apoptotic and inflammatory signaling. Signaling induced by cytotoxic forms of NO is involved in cardiac remodeling, controlling the coordinated increase in cardiomyocyte volume, mitochondrial biogenesis, and capillary density in CHF. The role of oxidized thiols and S-nitrosothiols in the mechanisms of apoptosis initiation has been identified. Also of interest in this regard are transnitrosylation reactions with varying concentrations of glutathione and glutaredoxin. Transnitrosylation reactions are involved in the transfer of active forms of NO to the SH groups of low-molecular thiol compounds (glutathione, cysteine) of signaling proteins, changing or suppressing their function, which leads to the initiation of apoptosis [41,42,43,44,45,46,47]. Nitrosative stress occurs against the background of discoordination of the thiol-disulfide system, which we confirmed by a deficiency of reduced glutathione and downregulation of the key enzymes GR and GPX4. GPX4 is considered a unique antioxidant enzyme due to its ability to inhibit membrane lipid peroxidation and ultimately reduce lipid radical levels. Reduced expression of GPX4 leads to a deficiency of intramitochondrial GSH and increases ferroptosis and fibrosis. The severity of GPX4 deficiency correlates with transforming growth factor β, demonstrating high prognostic value for the recurrence of atrial fibrosis in CHF [48,49,50,51,52]. Intermediates of the thiol-disulfide system have transport properties in relation to NO, thereby increasing its bioavailability, and are also capable of significantly limiting the cytotoxicity of NO and its derivatives in relation to cardiac remodeling, interstitial fibrosis, disarray of myocardial muscle bundles, ensuring the implementation of endogenous cardioprotection mechanisms [16,53,54]. Pharmacological measures aimed at preserving NO, reducing its conversion to ONOO- under the influence of ROS, and increasing the activity of glutathione-dependent enzymes inhibit the initiating mechanisms of apoptosis and the progression of CHF. All of this theoretically justifies the use of modulators of the SH/NO coupled system as metabolic cardioprotective agents. Previous studies have shown that NO modulators can inhibit apoptosis by influencing the expression of caspase-3 or increasing the expression of the heat shock protein HSP_70_. Our studies have established that the introduction of reduced glutathione, glutaredoxin, and thiotriazoline into a suspension of rat hippocampal neurons under conditions of nitrosative stress induction in vitro led to the preservation of malate dehydrogenase activity, an increase in mitochondrial charge, a decrease in nitrotyrosine, limited the inhibitory effect of peroxynitrite on phosphorylation, and increased the content of HSP_70_ [46,55,56].

The biochemical and metabolic cardioprotective profile of the novel molecule Angiolin demonstrated in this study may be attributed to its modulatory action on the NO and thiol-disulfide pathways, which is inherently linked to its distinct chemical structure. We hypothesize that Angiolin possesses unique properties that promote NO bioavailability and its efficient utilization by increasing the reduced equivalents of the thiol-disulfide system. Angiolin’s most pronounced positive effect is observed in relation to the active mobilization of the glutathione unit of the thiol-disulfide system, specifically, by increasing the synthesis of reduced glutathione and the expression of GPX4, which is aimed at neutralizing the products of nitrosative stress. Furthermore, we hypothesize that Angiolin itself may act as a NO transporter, forming stable S-nitrosyl complexes with it, as its chemical structure can act as a spin trap. The cardioprotective effect of Angiolin in doxorubicin-induced CHF can also be explained by its direct mitoprotective effect associated with an increase in intramitochondrial GSH and GPX4 [55]. Compelling findings from earlier studies were obtained when evaluating the cardioprotective effects of Angiolin on cardiohemodynamic parameters in a rabbit model of acute myocardial ischemia, providing deeper insight into its anti-ischemic mechanism. Intravenous administration of Angiolin (50 mg/kg) to rabbits with acute myocardial ischemia resulted in a significant reduction in left ventricular dysfunction. This was evidenced by increased left ventricular stroke work index and left ventricular work index, decreased total peripheral resistance, and elevated cardiac output, as well as increased left ventricular pressure and systolic blood pressure within 20 min following ischemia induction. Furthermore, these earlier investigations demonstrated the favorable effect of Angiolin on electrocardiographic parameters in ischemic animals, specifically through a reduction in total ST-segment elevation [10,46]. Angiolin can regulate the expression of protective proteins and factors by increasing GSH concentrations and influencing GSH-dependent posttranslational mechanisms. Angiolin is capable of maintaining sufficient levels of antioxidant enzyme expression during ischemia and hypoxia, possibly due to the interruption of NO-dependent mechanisms that suppress the expression of these enzymes (Figure 1) [16,46].

The reactivity of Angiolin toward NO is determined by the functional capacities of both the cationic and anionic moieties of its molecule. Specifically, L-lysine interacts with NO via its ε-amino group, leading to the formation of the corresponding N-nitroso derivative. Concurrently, the anionic component of the compound appears to form S-nitroso derivatives. The interaction of both the anionic and cationic moieties with NO exhibits a synergistic character, which accounts for the pronounced pharmacodynamic effect of the investigated agent. Notably, these empirical findings are consistent with previously published computational calculations [57].

Such interactions protect NO from reaction with ROS and its subsequent conversion into peroxynitrite, thereby interrupting nitrosative stress and mitigating its deleterious effects on myocardial tissue. Furthermore, Angiolin increases intra-mitochondrial and cytosolic levels of GSH and GPX4 expression. This not only reinforces cellular antioxidant defense but also regulates the expression of protective proteins and transcription factors by elevating GSH concentrations and influencing GSH-dependent post-translational mechanisms [55], alongside modulating eNOS and iNOS expression.

By restoring thiol-disulfide homeostasis, the GSH generated under Angiolin administration likely regulates key transcription factors, such as c-Jun and NF-κB, which display exceptionally high thiol-redox sensitivities [46]. Additionally, the synthesized GSH serves as an essential substrate for GPX4 to reduce membrane phospholipid hydroperoxides (PLOOH), thereby suppressing cell death via ferroptosis. As a result of these synergistic mechanisms, Angiolin produced a significant reduction in circulating cardio-specific biomarkers (sST2, NT-proBNP, and CK-MB) in animals with doxorubicin-induced CHF compared to other pharmacological agents, underscoring its high cardioprotective potential.

Mildronate (3-(2,2,2-trimethylhydrazine) propionate), widely used as a metabolic cardioprotector in post-Soviet countries, exerts its antihypoxic and cardioprotective effects through more economical use of free fatty acids via carnitine-dependent oxidation and their negative impact on mitochondria. Mildronate’s ability to influence acetylcholine receptors in cardiac endothelial cells by increasing γ-butyrobetaine has also been described, thereby indirectly activating total NOS and increasing NO production [58]. Mildronate can improve the quality of life of patients with CHF by increasing exercise tolerance and optimizing certain metabolic pathways [59,60]. We confirmed the cardioprotective activity of mildronate in a doxorubicin-induced CHF model. However, no effect of mildronate on the thiol-disulfide system or the myocardial NO system was observed in CHF. This study, along with a study after prenatal hypoxia, revealed that mildronate significantly increased iNOS mRNA expression and significantly decreased nitrotyrosine levels, further supporting its antioxidant properties [61].

Evaluating the obtained results, it can be assumed that the decrease in the content of nitrotyrosine and nitrites is due to the ability of NAC to inactivate ROS and cytotoxic forms of NO, possibly by forming nitrosothiols, as well as by reducing the expression of iNOS [62,63]. The decrease in iNOS expression under the influence of N-acetylcysteine can be explained not only by its ability to bind ROS involved in its expression, but also by the ability of glutathione formed from acetylcysteine to interrupt IL-1β-dependent mechanisms of expression of this enzyme [64]. Unlike other glutathione precursors, NAC readily penetrates mitochondria, especially in the presence of mitochondrial dysfunction, leading to an increase in intramitochondrial glutathione. By normalizing the functioning of the mitochondrial nitroxidergic system in brain mitochondria in CHF by limiting iNOS activity, NAC may regulate NO-dependent apoptotic mechanisms. The reduction in iNOS expression can also be explained by NAC’s ability to attenuate the activation of nuclear factor NF-κB, which controls cell cycle function [65,66,67]. NAC has also been shown to reduce cardiac fibrosis and associated ventricular hypertrophy in male neonates with maternal obesity, possibly by reducing nitrosative stress and restoring normal Akt-mTOR signaling in the offspring. NAC, by increasing oxidized glutathione levels in the myocardium and blood, can reduce cardiac hypertrophy and interstitial fibrosis and prevent left ventricular systolic dysfunction in transgenic rabbits [68,69].

Thiotriazoline produced a significant effect on NO and thiol-disulfide system parameters. In terms of its effect on the main parameters of these systems, as well as on specific molecular cardiac markers, it exceeded the effects of not only mildronate but also heptral and NAC. This effect of Thiotriazoline is related to its structure, namely thiazate, which is a specific scavenger of cytotoxic NO derivatives and inhibits ROS overproduction by mitochondrial bioenergetic systems. Furthermore, thiazate reduces iNOS expression, possibly by interrupting ROS-dependent mechanisms of IL-1β and TNF-α expression. Thiotriazoline, a powerful antioxidant, can activate sirtuin 1–3 genes (deacetylating mitochondrial proteins that regulate ATP production and eNOS). Thiotriazoline can regulate the activation of nuclear factor NF-κB and thereby inhibit apoptosis [10].

Thiotriazoline, due to its properties as a NO scavenger, is able to influence the expression of the transcription factor NF-κB by regulating the dynamic SH/SS ratio in ROS-sensitive proteins—Cys 252, 154, and 61—in the DNA-binding domains of NF-κB. Thiotriazoline can restore these groups during reversible ROS-dependent inactivation. Thiotriazoline increases the expression of Red/Oxi-sensitive genes during ischemia and hypoxia, leading to increased synthesis of both antioxidant defense enzymes (GPX4, GR) and key enzymes of energy compensatory shunts (NAD-dependent malate dehydrogenase, etc.) [70,71].

Thiotriazoline enhances the NO-modulating efficacy of L-arginine when co-administered, thereby increasing NO synthesis, transport, and bioavailability while potentiating cardioprotective and endothelioprotective actions [72]. A study involving 8298 patients with various cardiovascular diseases, including 5700 patients with various types of coronary heart disease, found that the administration of thiotriazoline resulted in a significant reduction in the incidence of ventricular arrhythmias and correction of rhythm disturbances, improved myocardial metabolism, and increased exercise tolerance (Clinical trial reports Polivoda 2006; Dzyak 2010; Zupanets & Podpruzhnikov, 2011) [10,19,46]. The observed effect of heptral may be due to the fact that, firstly, S-adenosylmethionine, by promoting the synthesis of glutathione, creates conditions for the conjugation of free radical reaction products, in particular ROS, with glutathione and their inactivation [73], and secondly, S-adenosylmethionine serves as an effector molecule for riboswitches (RNA elements that control gene expression through metabolite-induced changes in their secondary structure) [74]. The antioxidant action of adenosylmethionine, aimed at reducing homocysteine and increasing glutathione, also determines its cardioprotective activity [75]. By binding to S-adenosylmethionine, riboswitches form a termination structure that stops transcription and inhibits mRNA synthesis [76].

## 5. Conclusions

Based on comprehensive molecular and biochemical studies of the NO system, nitrosative stress, and the glutathione branch of the thiol-disulfide system in rats with experimental doxorubicin-induced CHF, we have obtained new data on the coupling of the NO system and the thiol-disulfide system, which are involved in mechanisms of cardioprotection and cardiac destruction. The experimental data indicate that the severity of myocardial damage in CHF (based on NT-proBNP, sST2, and CK-MB markers) and nitrosative stress depend on the homeostasis of the glutathione system (GR activity, GPX4 protein expression, and GSH levels), as well as eNOS/iNOS expression NT-proBNP, sST2, and CK-MB are established cardiac biomarkers used to assess myocardial status in ischemia and heart failure. International practice guidelines recommend their quantification in blood for disease diagnosis and monitoring, as well as for evaluating hemodynamic wall stress, myocardial fibrosis/inflammation, and tissue injury [77,78]. Pharmacological modulation of the coupled SH/NO systems in CHF leads to the normalization of the NO and glutathione systems, a reduction in nitrosative stress, and a potential enhancement of cardioprotection. The experimental findings reveal the potential of pharmacological modulators of the SH and NO systems as metabolitotropic cardioprotectors for heart failure. Based on the conducted studies, the feasibility of further preclinical study of Angiolin as a promising cardioprotective agent for CHF is experimentally substantiated. Furthermore, the obtained results experimentally substantiate the further in-depth preclinical study of the effects of Thiotriazoline, Heptral, and NAC on the molecular and biochemical mechanisms of myocardial injury in CHF and following the administration of anthracycline antineoplastic agents.

**Study Limitations**

Several key limitations should be noted:

1. Limited time period

The protective effects were studied for 30 days after the onset of doxorubicin-induced CHF after administration of pharmacological agents. Long-term effects after discontinuation of pharmacological agents were not studied.

**Limitations of the Chosen Model**

The doxorubicin-induced animal model of heart failure does not fully replicate critical components of classical CHF, such as pronounced focal fibrosis and marked compensatory myocardial hypertrophy resulting from pressure overload. Consequently, this experimental CHF model poorly recapitulates certain pathological target pathways modulated by the renin–angiotensin system and may exhibit low sensitivity to agents belonging to this therapeutic class. For in-depth preclinical evaluation of potential drug candidates, further studies are required using animal models of CHF induced by left coronary artery occlusion or left coronary artery occlusion combined with exhaustive physical exercise.

**Statistical Limitations**

We included 10 animals per group, which is a relatively small sample size. Nevertheless, given the mortality rate, this number of animals is sufficient to detect reliable pharmacological effects. However, this number of animals may be insufficient to study more intimate mechanisms of pharmacodynamics and toxicodynamics.

**Limitations Related to the Mechanism of Action**

Incomplete pathway analysis: The study focused on individual molecular markers (eNOS, iNOS, nitrotyrosine, NT-proBNP, sST2 and CK-MB, GSH, GPX4, GR), but did not comprehensively examine all potential mechanisms of CHF pathogenesis and the cardioprotective effects of pharmacological agents (cardiac immunohistochemistry to detect eNOS-positive, iNOS-positive, GPX-positive, and GR-positive cells). In vitro and in vivo studies were performed with glutathione depletion and/or selective inhibition of NOS isoforms.

**Limitations Related to Translational Medicine**

**Sexual Dimorphism**

Only male animals were used in the experiment, and potential sex differences in drug response were not taken into account, which may limit the applicability of the obtained results.

**Species-Specific Limitations**

The use of rats alone limits extrapolation to humans without additional studies in other animal species or human cell models.

**Age Limitations**

The study used adult rats aged 2.5 months, but did not examine the effects of pharmacological agents on cardiac aging in older rats, which may have varying sensitivities to doxorubicin, angiolin, thiotriazoline, heptral, NAC, and mildronate.

**Prospects for Further Research**

Further research should focus on the study of SH/NO-dependent mechanisms of cardioprotection in CHF and their correlation with survival, cardiac bioelectrical activity, and cardiac hemodynamics. It is necessary to conduct a correlation analysis of the obtained molecular and biochemical parameters and identify adequate criteria for assessing disturbances in the fine links of myocardial metabolism in CHF for use in the targeted selection of metabolic cardioprotective agents. The importance of combination regimens including SH/NO modulators and Krebs cycle intermediates, compensatory energy pathway intermediates, and modulators of endogenous cytoprotective and cardioprotective factors should be emphasized. To enhance cardioprotection, it is necessary to address all molecular and biochemical mechanisms involved in myocardial damage in CHF. The most rational approach would be a combination of agents acting on multiple target links. These findings provide a theoretical foundation for developing rational multi-target combinations of metabolic cardioprotective agents.

## Figures and Tables

**Figure 1:**
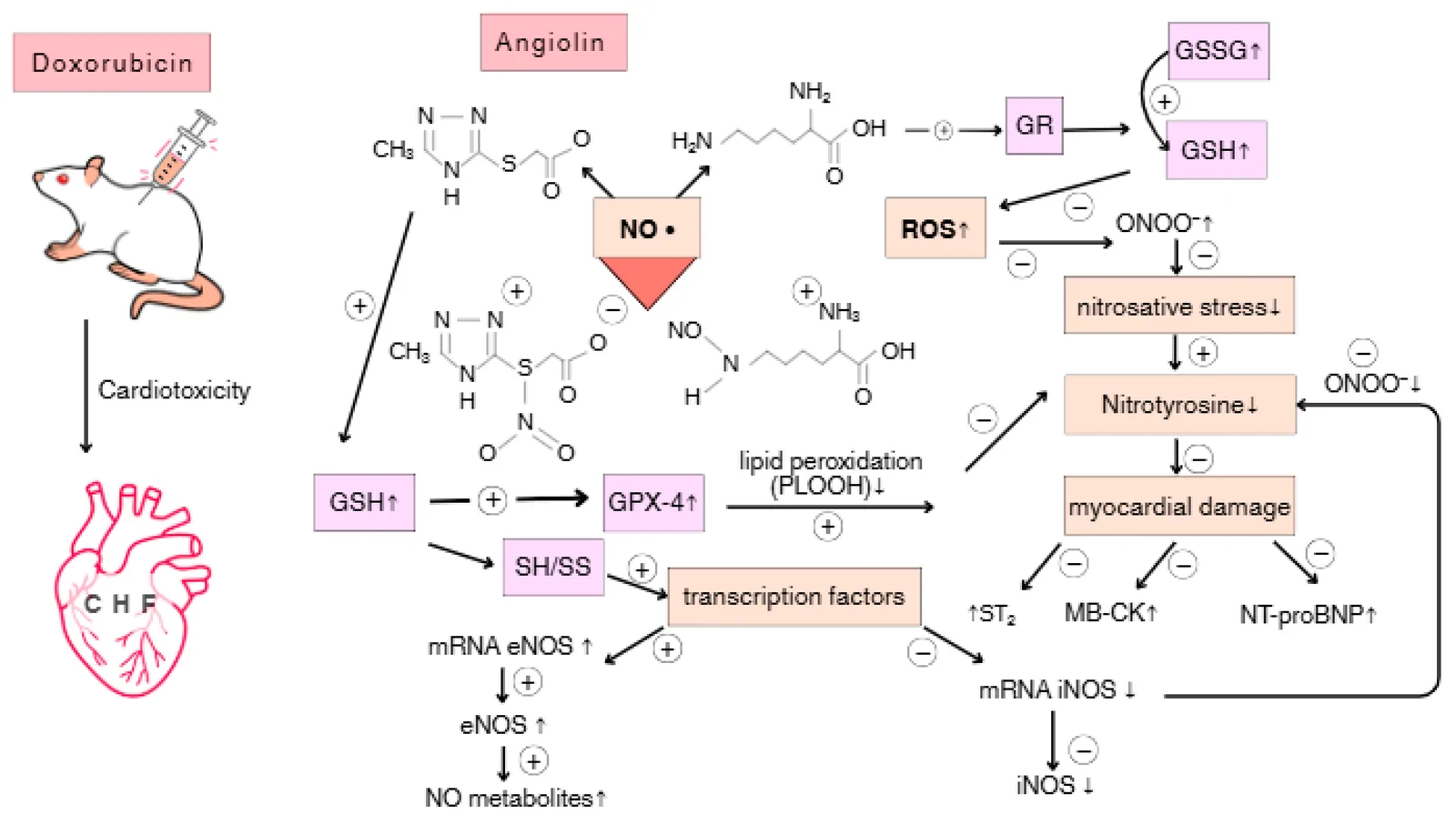
Hypothetical mechanism of the cardioprotective action of (S)-2,6-diaminohexanoic acid 3-methyl-1,2,4-triazolyl-5-thioacetate (Angiolin) through the modulation of coupled NO and thiol-disulfide systems in the myocardium during experimental doxorubicin-induced CHF. The symbols (+) and (−) indicate activation and inhibition, respectively.

**Table 1:** Concentration of molecular and biochemical markers in the blood of animals with doxorubicin-induced CHF (mean ± standard deviation (SD)).

Experimental Groups (n = 10)	CK-MB, IU/L	NT-proBNP pg/mL	sST2, ng/mL
Intact	21.2 ± 2.1	203.3 ± 11.0	20.4 ± 0.7
Control (CHF)	47.9 ± 3.2 ^1^	712.2 ± 15.6 ^1^	105.2 ± 7.5
CHF + NAC	33.2 ± 2.1* ^1^	411.5 ± 18.4 * ^1^ #	51.7 ± 2.6 * ^1^ #
CHF + Heptral	37.7 ± 2.2 * ^1^	518.0 ± 21.1 * ^1^	77.5 ± 4.8 * ^1^
CHF + Angiolin	25.3 ± 1.7 * ^1^ #	287.3 ± 11.4 * ^1^ #	28.5 ± 1.0 * #
CHF + Thiotriazoline	30.1 ± 2.5 * ^1^ #	324.2 ± 15.2 * ^1^ #	40.2 ± 5.0 * ^1^ #
CHF + Mildronate	37.8 ± 3.1 * ^1^	483.0 ± 17.2 * ^1^	78.3 ± 4.1 ^1^ *

Notes: *—*p* ≤ 0.05 in relation to the control group of animals; ^1^—*p* ≤ 0.05 in relation to the intact group of animals; #—*p* ≤ 0.05 compared to the CHF + mildronate group.

**Table 2:** Indicators of the nitroxidergic system and nitrosative stress in the cytosolic fraction of the heart homogenate of experimental animals with doxorubicin-induced CHF (mean ± standard deviation (SD)).

Experimental Groups (n = 10)	eNOS, pg/mL	NO Metabolites (NOx), µmol/L	Nitrotyrosine, pg/mL	iNOS, pg/mL
Intact	58.8 ± 3.2	5.7 ± 0.32	18.8 ± 1.4	19.5 ± 1.7
Control (CHF)	21.2 ± 2.2 ^1^	3.4± 0.21 ^1^	131.4 ± 15.0 ^1^	88.2 ± 6.33 ^1^
CHF + NAC	37.5 ± 2.4 * ^1^ #	4.6 ± 0.37 * ^1^ #	62.2 ± 4.8 * ^1^ #	54.0 ± 4.2 * ^1^ #
CHF + Heptral	30.4 ± 3.4 * ^1^ #	3.98 ± 0.12 * ^1^	77.2 ± 8.1 * ^1^ #	70.0 ± 9.1 * ^1^
CHF + Angiolin	57.5 ± 3.3 * #	5.4 ± 0.23 * #	38.3 ± 4.1 * ^1^ #	38.2 ± 3.0 * #
CHF + Thiotriazoline	41.2 ± 3.3 * #	4.8 ± 0.18 * ^1^#	45.2 ± 3.1 * ^1^	42.7 ± 3.2 * ^1^ #
CHF + Mildronate	21.6 ± 3.2 ^1^	3.6 ± 1.2 ^1^	99.7 ± 8.7 * ^1^	89.3 ± 10.2 ^1^

Notes: *—*p* ≤ 0.05 in relation to the control group of animals; ^1^—*p* ≤ 0.05 in relation to the intact group of animals; #—*p* ≤ 0.05 compared to the CHF + mildronate group.

**Table 3:** Indicators of the nitroxidergic system and nitrosative stress in the mitochondrial fraction of the heart homogenate of experimental animals with doxorubicin-induced CHF (mean ± standard deviation (SD)).

Experimental Groups (n = 10)	eNOS, pg/mL	NO Metabolites (NOx), µmol/L	Nitrotyrosine, pg/mL	iNOS, pg/mL
Intact	3.8 ± 0.2	2.3 ± 0.11	7.74 ± 0.62	8.2 ± 0.71
Control (CHF)	2.2 ± 0.2 ^1^	1.4 ± 0.10 ^1^	33.7 ± 4.1 ^1^	23.2 ± 2.11 ^1^
CHF + NAC	3.11 ± 0.22 * ^1^ #	2.00 ± 0.11 * ^1^ #	15.4 ± 1.51 * ^1^ #	17.2 ± 1.4 * ^1^ #
CHF + Heptral	2.1 ± 0.41 ^1^	1.67 ± 0.22 ^1^	28.7 ± 1.55 * ^1^ #	24.7 ± 2.3 ^1^
CHF + Angiolin	3.85 ± 0.21 * #	2.21 ± 0.12 * #	9.2 ± 0.18 * ^1^ #	9.5 ±0.41 * ^1^ #
CHF + Thiotriazoline	3.17 ± 0.11 * ^1^ #	2.12 ± 0.15 * #	12.3 ± 1.42 * ^1^ #	11.2 ±0.55 * ^1^ #
CHF + Mildronate	2.2 ± 0.37 ^1^	1.27 ± 0.3 ^1^	31.8 ± 5.12 ^1^	23.1 ± 3.7 ^1^

Notes: *—*p* ≤ 0.05 in relation to the control group of animals; ^1^—*p* ≤ 0.05 in relation to the intact group of animals; #—*p* ≤ 0.05 compared to the CHF + mildronate group.

**Table 4:** Expression of eNOS mRNA and iNOS mRNA in experimental animals with doxorubicin-induced CHF (mean ± standard deviation (SD)).

Experimental Groups (n = 10)	eNOS mRNA, a.u.	iNOS mRNA, a.u.
Intact	1.000 ± 0.0012	1.000 ± 0.001
Control (CHF)	0.11 ± 0.00015 ^1^	7.88 ± 0.004 ^1^
CHF + NAC	0.58 ± 0.00011 ^1^ *	3.88 ± 0.0072 ^1^ * #
CHF + Heptral	0.20 ± 0.0005 ^1^ * #	4.68 ± 0.0012 ^1^ * #
CHF + Angiolin	2.17 ± 0.0014 ^1^ * #	1.88 ± 0.0014 ^1^ * #
CHF + Thiotriazoline	1.16 ± 0.0012 ^1^ * #	2.55 ± 0.0007 ^1^ * #
CHF + Mildronate	0.11 ± 0.0017 ^1^	8.72 ± 0.0052 * ^1^

Notes: *—*p* ≤ 0.05 in relation to the control group of animals; ^1^—*p* ≤ 0.05 in relation to the intact group of animals; #—*p* ≤ 0.05 compared to the CHF + mildronate group.

**Table 5:** Indicators of the glutathione branch of the thiol-disulfide system in the cytosolic fraction of the heart of animals with doxorubicin-induced CHF (mean ± standard deviation (SD)).

Experimental Groups (n = 10)	GPX-4, pg/mL	GSH, µmol/g Tissue	GSSG, µmol/g Tissue	GR, µmol/mg/min
Intact	62.7 ±4.8	3.7 ± 0.21	0.11 ± 0.02	27.7 ± 3.43
Control (CHF)	28.8 ± 2.2 ^1^	0.97 ± 0.08 ^1^	0.32 ± 0.01 ^1^	8.9 ± 0.77 ^1^
CHF + NAC	38.6 ± 4.5 ^1^ * #	1.57 ± 0.08 * ^1^ #	0.22 ± 0.01 * ^1^ #	14.8 ± 0.82 * ^1^ #
CHF + Heptral	42.8 ± 2.32 ^1^ * #	1.28± 0.07 * ^1^ #	0.23 ± 0.01 * ^1^ #	12.6 ± 0.55 ^1^ * #
CHF + Angiolin	54.8 ± 3.47 * ^1^ #	2.8± 0.11 * ^1^ #	0.17 ± 0.02 * ^1^ #	18.6 ± 1.10 * ^1^ #
CHF + Thiotriazoline	46.2 ± 3.77 * ^1^ #	1.72± 1.10 * ^1^ #	0.187 ± 0.03 * ^1^ #	15.2 ± 0.77 * ^1^ #
CHF + Mildronate	29.91 ± 3.38 ^1^	0.94± 0.09 ^1^	0.31 ± 0.01 ^1^	9.8 ± 0.56 ^1^

Notes: *—*p* ≤ 0.05 in relation to the control group of animals; ^1^—*p* ≤ 0.05 in relation to the intact group of animals; #—*p* ≤ 0.05 compared to the CHF + mildronate group.

**Table 6:** Indicators of the glutathione branch of the thiol-disulfide system in the mitochondrial fraction of the heart of animals with doxorubicin-induced CHF (mean ± standard deviation (SD)).

Experimental Groups (n = 10)	GPX-4, pg/mL	GSH, µmol/g Tissue	GSSG, µmol/g Tissue	GR, µmol/mg/min
Intact	34.5 ± 2.3	0.83 ± 0.018	0.028 ± 0.001	11.2 ± 1.23
Control (CHF)	11.2 ± 1.00 ^1^	0.188 ± 0.022 ^1^	0.072 ± 0.001 ^1^	3.8 ± 0.25 ^1^
CHF + NAC	21.2 ± 1.12 ^1^ * #	0.33 ± 0.042 * ^1^	0.054 ± 0.002 * ^1^	6.7 ± 0.32 * ^1^ #
CHF + Heptral	17.3 ± 0.75 ^1^ * #	0.192 ± 0.022 ^1^	0.066 ± 0.01 ^1^	3.9 ± 0.35 ^1^
CHF + Angiolin	28.3 ± 1.00 * ^1^	0.52 ± 0.032 * ^1^ #	0.038 ± 0.001 * ^1^ #	8.3 ± 0.21 * ^1^ #
CHF + Thiotriazoline	22.8 ± 0.87 * ^1^	0.39 ± 0.014 * ^1^	0.049 ± 0.003 * ^1^ #	7.5 ± 0.37 * ^1^ #
CHF + Mildronate	11.9 ± 0.92 ^1^	0.34 ± 0.042 ^1^ *	0.069 ± 0.007 ^1^	3.2 ± 0.42 ^1^

Notes: *—*p* ≤ 0.05 in relation to the control group of animals; ^1^—*p* ≤ 0.0*5* in relation to the intact group of animals; #—*p* ≤ 0.05 compared to the CHF + mildronate group.

## Data Availability

The raw data generated during the current study are available from the corresponding author upon reasonable request and with permission from the administration of the institution where the research was conducted.

## Supplementary Materials

The following supporting information can be downloaded at: https://www.mdpi.com/article/10.3390/metabo16100715/s1.
