# Supplementary material for: Cardioprotective Effects of Coupled SH/NO System Modulators in a Rat Model of Doxorubicin-Induced Heart Failure
Source: Metabolites. 2026 Sep 25;16(10):715. doi: 10.3390/metabo16100715 (PMC13707050; doi:10.3390/metabo16100715)
Supplement: Supplementary file 1 [file metabolites-16-00715-s001.zip › metabolites-4583603-supplementary.pdf]

Supplementary Materials

Cardioprotective Effects of Coupled SH/NO System Modulators in a Rat Model of Doxorubicin-Induced Heart Failure

```
  for (i in 1:(k - 1)) {
    for (j in (i + 1):k) {
      test <- tsum.test(mean.x = m_vec[i], s.x = sd_vec[i], n.x = n,
                        mean.y = m_vec[j], s.y = sd_vec[j], n.y = n)

      сравнение <- paste(gr_names[i], "vs", gr_names[j])
      res_row <- data.frame(Comparison = comparison, P_raw = test$p.value)
      results <- rbind(results, res_row)
    }
  }

  results$P_Bonferroni <- p.adjust(results$P_raw, method = "bonferroni")
  results$P_Holm <- p.adjust(results$P_raw, method = "holm")

  return(results)
}

my_groups <- c("CHF+ NAC", "CHF+ Heptral", "CHF+ Angiolin", "CHF+ Thiotriazoline", "Mildronate" )
means <- c(4.6, 3.98, 5.4, 4.8, 3.6)
sds <- c(0.37, 0.12, 0.23, 0.18, 1.21)
n_sample <- 30

final_table <- all_function(my_groups, means, sds, n_sample)
print(final_table)
```

Comparison	P_raw	P_Bonferroni	P_Holm
CHF+ NAC vs CHF+ Heptral	2.588386e-10	2.588386e-09	1.553032e-09
CHF+ NAC vs CHF+ Angiolin	1.865531e-13	1.865531e-12	1.305872e-12
CHF+ NAC vs CHF+ Thiotriazoline	1.095138e-02	1.095138e-01	2.190275e-02
CHF+ NAC vs Mildronate	1.228444e-04	1.228444e-03	3.685333e-04
CHF+ Heptral vs CHF+ Angiolin	8.821551e-31	8.821551e-30	8.821551e-30
CHF+ Heptral vs CHF+ Thiotriazoline	2.165200e-26	2.165200e-25	1.948680e-25
CHF+ Heptral vs Mildronate	9.741860e-02	9.741860e-01	9.741860e-02
CHF+ Angiolin vs CHF+ Thiotriazoline	7.192693e-16	7.192693e-15	5.754154e-15
CHF+ Angiolin vs Mildronate	4.775518e-09	4.775518e-08	2.387759e-08
CHF+ Thiotriazoline vs Mildronate	7.903940e-06	7.903940e-05	3.161576e-05

Figure S1. Analysis output in R software package
